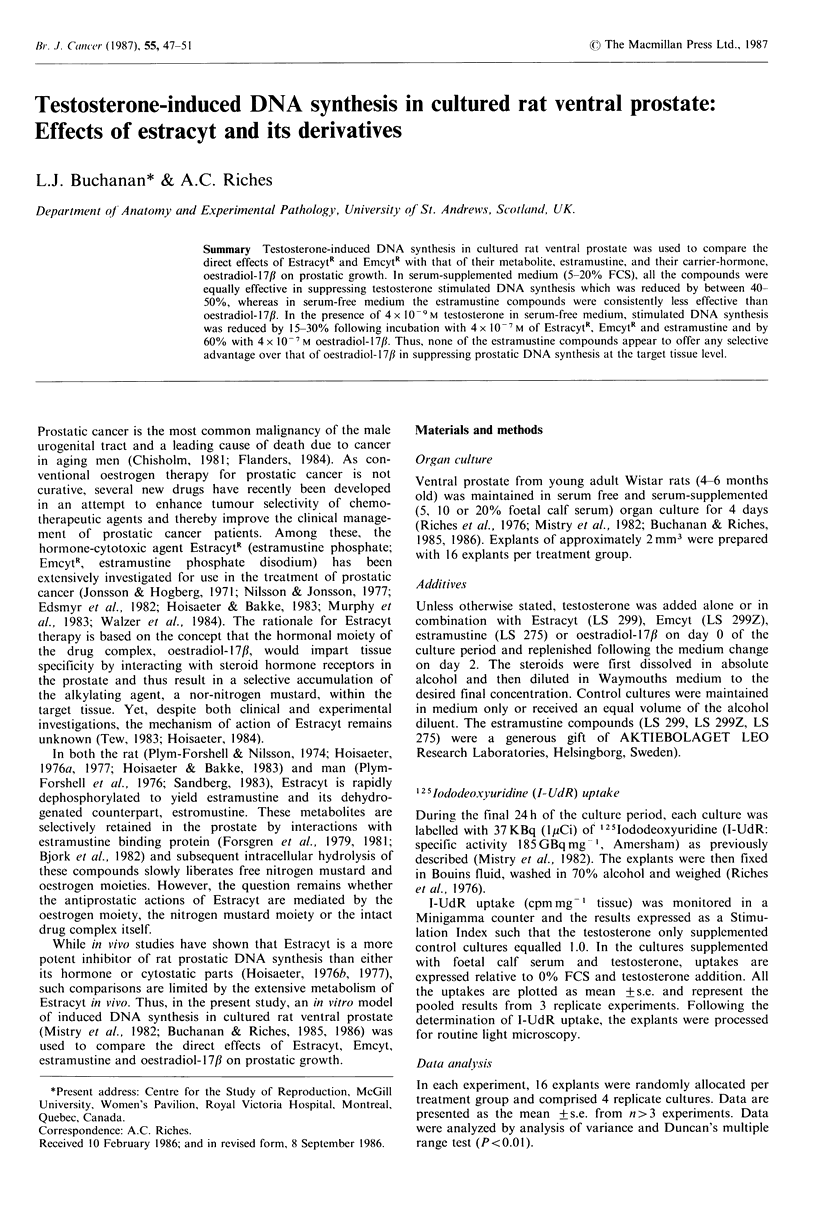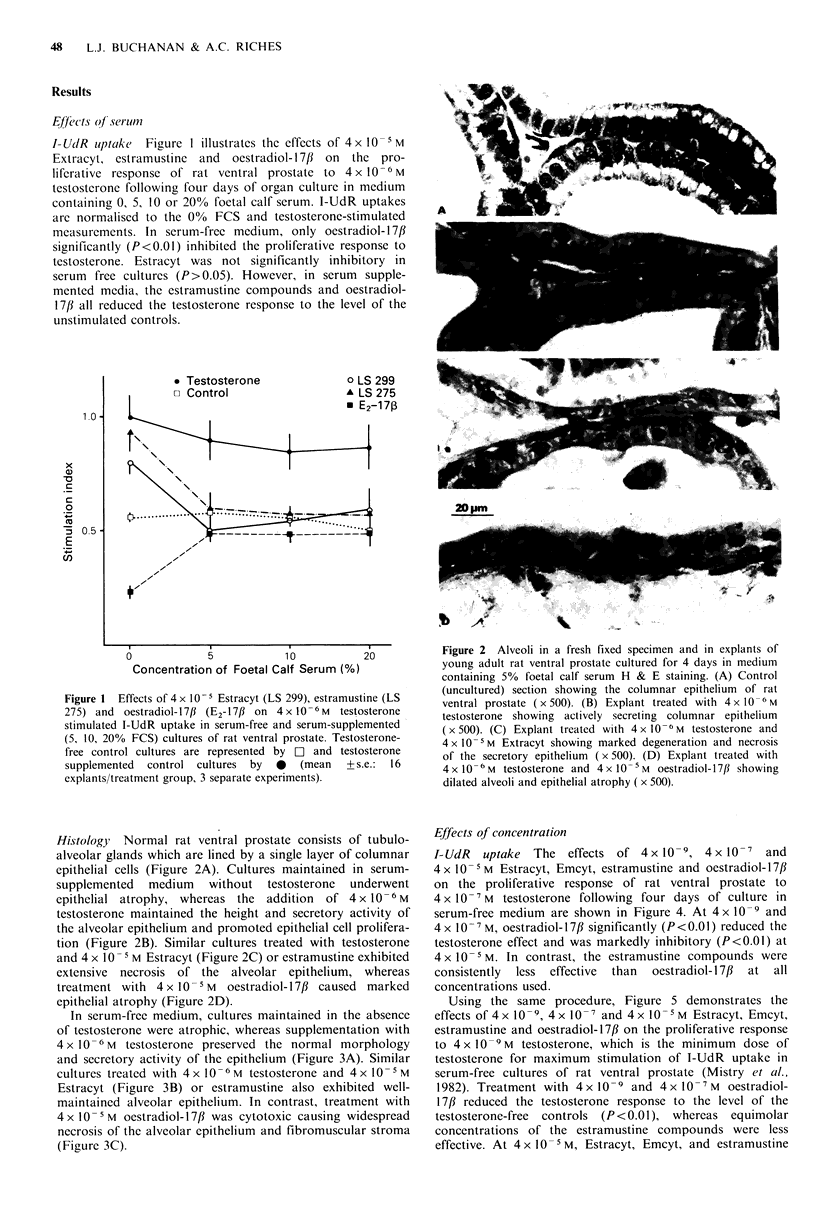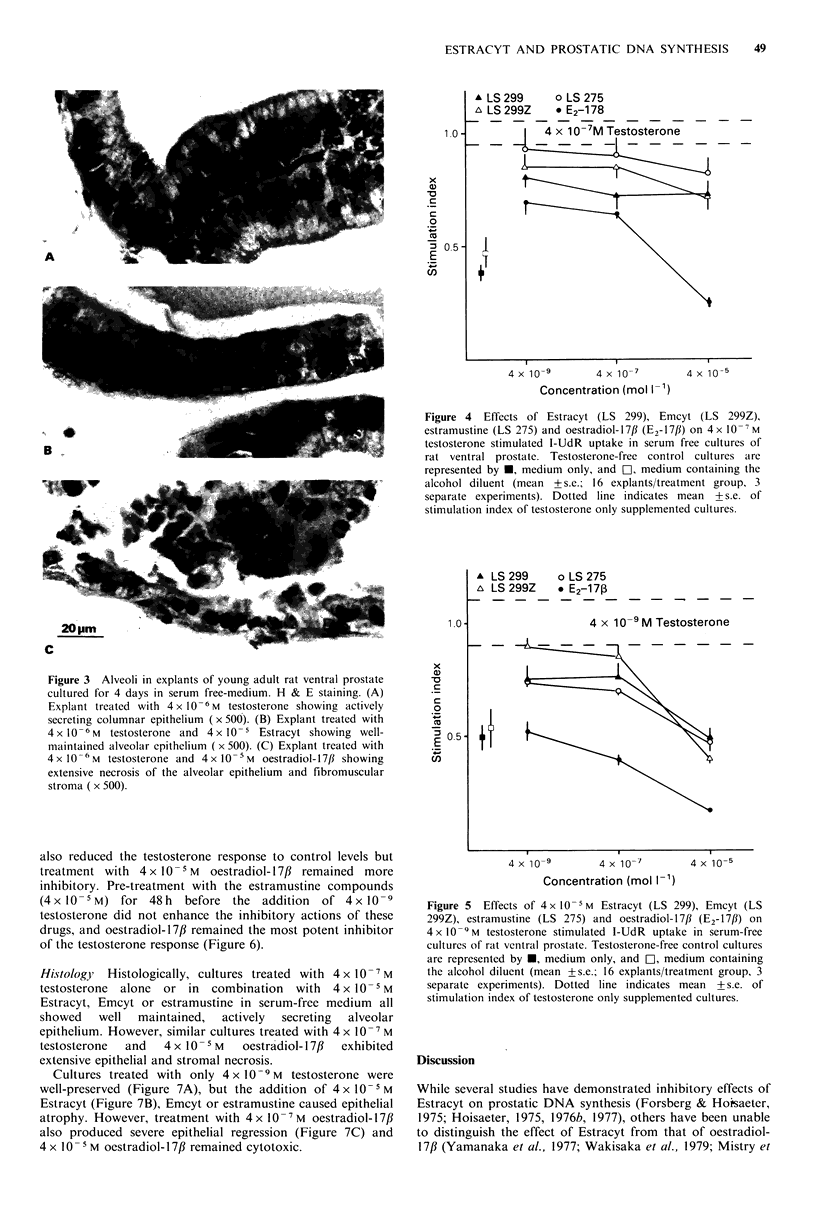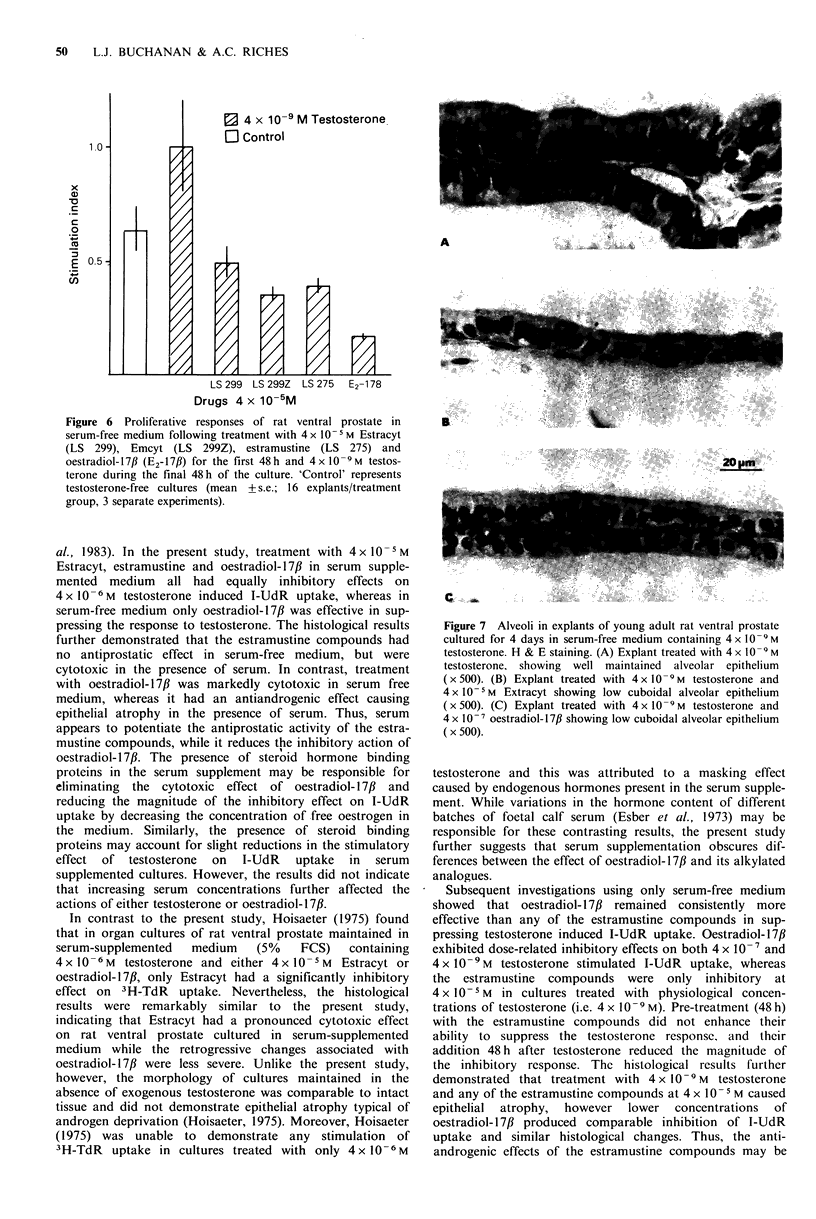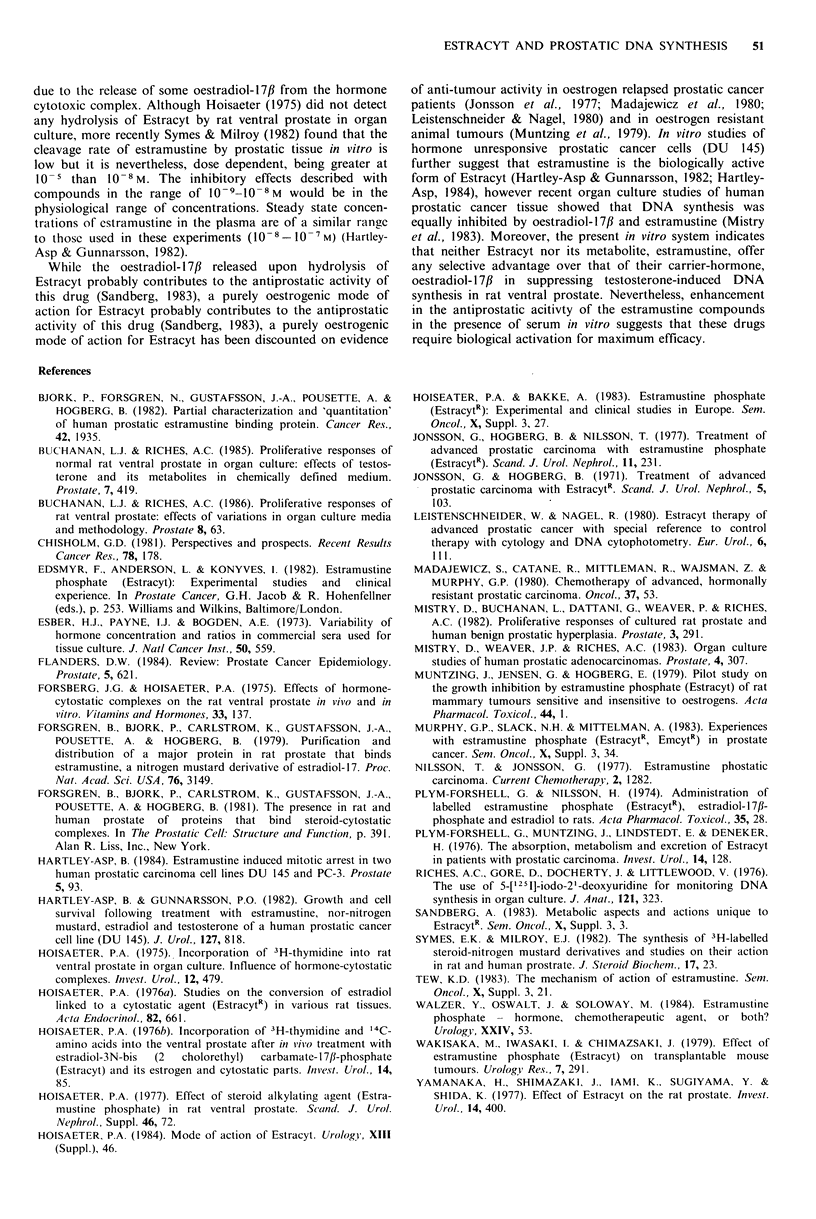# Testosterone-induced DNA synthesis in cultured rat ventral prostate: effects of estracyt and its derivatives.

**DOI:** 10.1038/bjc.1987.10

**Published:** 1987-01

**Authors:** L. J. Buchanan, A. C. Riches

## Abstract

**Images:**


Br. J. Cancer (1987), 55, 47–51

© The Macmillan Press Ltd., 1987

### Testosterone-induced DNA synthesis in cultured rat ventral prostate: Effects of estracyt and its derivatives

L.J. Buchanan* & A.C. Riches

*Department of Anatomy and Experimental Pathology, University of St. Andrews, Scotland, UK.*

**Summary** Testosterone-induced DNA synthesis in cultured rat ventral prostate was used to compare the direct effects of Estracyt[R] and Emcyt[R] with that of their metabolite, estramustine, and their carrier-hormone, oestradiol-17$\beta$ on prostatic growth. In serum-supplemented medium (5–20% FCS), all the compounds were equally effective in suppressing testosterone stimulated DNA synthesis which was reduced by between 40–50%, whereas in serum-free medium the estramustine compounds were consistently less effective than oestradiol-17$\beta$. In the presence of $4\times10^{-9}$ M testosterone in serum-free medium, stimulated DNA synthesis was reduced by 15–30% following incubation with $4\times10^{-7}$ M of Estracyt[R], Emcyt[R] and estramustine and by 60% with $4\times10^{-7}$ M oestradiol-17$\beta$. Thus, none of the estramustine compounds appear to offer any selective advantage over that of oestradiol-17$\beta$ in suppressing prostatic DNA synthesis at the target tissue level.

Prostatic cancer is the most common malignancy of the male urogenital tract and a leading cause of death due to cancer in aging men (Chisholm, 1981; Flanders, 1984). As conventional oestrogen therapy for prostatic cancer is not curative, several new drugs have recently been developed in an attempt to enhance tumour selectivity of chemotherapeutic agents and thereby improve the clinical management of prostatic cancer patients. Among these, the hormone-cytotoxic agent Estracyt[R] (estramustine phosphate; Emcyt[R], estramustine phosphate disodium) has been extensively investigated for use in the treatment of prostatic cancer (Jonsson & Hogberg, 1971; Nilsson & Jonsson, 1977; Edsmyr *et al.*, 1982; Hoisaeter & Bakke, 1983; Murphy *et al.*, 1983; Walzer *et al.*, 1984). The rationale for Estracyt therapy is based on the concept that the hormonal moiety of the drug complex, oestradiol-17$\beta$, would impart tissue specificity by interacting with steroid hormone receptors in the prostate and thus result in a selective accumulation of the alkylating agent, a nor-nitrogen mustard, within the target tissue. Yet, despite both clinical and experimental investigations, the mechanism of action of Estracyt remains unknown (Tew, 1983; Hoisaeter, 1984).

In both the rat (Plym-Forshell & Nilsson, 1974; Hoisaeter, 1976*a*, 1977; Hoisaeter & Bakke, 1983) and man (Plym-Forshell *et al.*, 1976; Sandberg, 1983), Estracyt is rapidly dephosphorylated to yield estramustine and its dehydrogenated counterpart, estromustine. These metabolites are selectively retained in the prostate by interactions with estramustine binding protein (Forsgren *et al.*, 1979, 1981; Bjork *et al.*, 1982) and subsequent intracellular hydrolysis of these compounds slowly liberates free nitrogen mustard and oestrogen moieties. However, the question remains whether the antiprostatic actions of Estracyt are mediated by the oestrogen moiety, the nitrogen mustard moiety or the intact drug complex itself.

While *in vivo* studies have shown that Estracyt is a more potent inhibitor of rat prostatic DNA synthesis than either its hormone or cytostatic parts (Hoisaeter, 1976*b*, 1977), such comparisons are limited by the extensive metabolism of Estracyt *in vivo*. Thus, in the present study, an *in vitro* model of induced DNA synthesis in cultured rat ventral prostate (Mistry *et al.*, 1982; Buchanan & Riches, 1985, 1986) was used to compare the direct effects of Estracyt, Emcyt, estramustine and oestradiol-17$\beta$ on prostatic growth.

*Present address: Centre for the Study of Reproduction, McGill University, Women's Pavilion, Royal Victoria Hospital, Montreal, Quebec, Canada.
Correspondence: A.C. Riches.
Received 10 February 1986; and in revised form, 8 September 1986.

### Materials and methods

### *Organ culture*

Ventral prostate from young adult Wistar rats (4–6 months old) was maintained in serum free and serum-supplemented (5, 10 or 20% foetal calf serum) organ culture for 4 days (Riches *et al.*, 1976; Mistry *et al.*, 1982; Buchanan & Riches, 1985, 1986). Explants of approximately 2 $mm^3$ were prepared with 16 explants per treatment group.

### *Additives*

Unless otherwise stated, testosterone was added alone or in combination with Estracyt (LS 299), Emcyt (LS 299Z), estramustine (LS 275) or oestradiol-17$\beta$ on day 0 of the culture period and replenished following the medium change on day 2. The steroids were first dissolved in absolute alcohol and then diluted in Waymouths medium to the desired final concentration. Control cultures were maintained in medium only or received an equal volume of the alcohol diluent. The estramustine compounds (LS 299, LS 299Z, LS 275) were a generous gift of AKTIEBOLAGET LEO Research Laboratories, Helsingborg, Sweden).

### *$^{125}$Iododeoxyuridine (I-UdR) uptake*

During the final 24 h of the culture period, each culture was labelled with 37 KBq (1 $\mu$Ci) of $^{125}$Iododeoxyuridine (I-UdR: specific activity 185 GBq $mg^{-1}$, Amersham) as previously described (Mistry *et al.*, 1982). The explants were then fixed in Bouins fluid, washed in 70% alcohol and weighed (Riches *et al.*, 1976).

I-UdR uptake (cpm $mg^{-1}$ tissue) was monitored in a Minigamma counter and the results expressed as a Stimulation Index such that the testosterone only supplemented control cultures equalled 1.0. In the cultures supplemented with foetal calf serum and testosterone, uptakes are expressed relative to 0% FCS and testosterone addition. All the uptakes are plotted as mean $\pm$s.e. and represent the pooled results from 3 replicate experiments. Following the determination of I-UdR uptake, the explants were processed for routine light microscopy.

### *Data analysis*

In each experiment, 16 explants were randomly allocated per treatment group and comprised 4 replicate cultures. Data are presented as the mean $\pm$s.e. from $n>3$ experiments. Data were analyzed by analysis of variance and Duncan's multiple range test ($P<0.01$).

### Results

*Effects of serum*

*I-UdR uptake* Figure 1 illustrates the effects of $4\times10^{-5}$ M Extracyt, estramustine and oestradiol-17$\beta$ on the proliferative response of rat ventral prostate to $4\times10^{-6}$ M testosterone following four days of organ culture in medium containing 0, 5, 10 or 20% foetal calf serum. I-UdR uptakes are normalised to the 0% FCS and testosterone-stimulated measurements. In serum-free medium, only oestradiol-17$\beta$ significantly ($P<0.01$) inhibited the proliferative response to testosterone. Estracyt was not significantly inhibitory in serum free cultures ($P>0.05$). However, in serum supplemented media, the estramustine compounds and oestradiol-17$\beta$ all reduced the testosterone response to the level of the unstimulated controls.

**Figure 1** Effects of $4\times10^{-5}$ Estracyt (LS 299), estramustine (LS 275) and oestradiol-17$\beta$ ($E_2$-17$\beta$ on $4\times10^{-6}$ M testosterone stimulated I-UdR uptake in serum-free and serum-supplemented (5, 10, 20% FCS) cultures of rat ventral prostate. Testosterone-free control cultures are represented by □ and testosterone supplemented control cultures by ● (mean ±s.e.: 16 explants/treatment group, 3 separate experiments).

**Figure 2** Alveoli in a fresh fixed specimen and in explants of young adult rat ventral prostate cultured for 4 days in medium containing 5% foetal calf serum H & E staining. (A) Control (uncultured) section showing the columnar epithelium of rat ventral prostate (×500). (B) Explant treated with $4\times10^{-6}$ M testosterone showing actively secreting columnar epithelium (×500). (C) Explant treated with $4\times10^{-6}$ M testosterone and $4\times10^{-5}$ M Extracyt showing marked degeneration and necrosis of the secretory epithelium (×500). (D) Explant treated with $4\times10^{-6}$ M testosterone and $4\times10^{-5}$ M oestradiol-17$\beta$ showing dilated alveoli and epithelial atrophy (×500).

*Histology* Normal rat ventral prostate consists of tubulo-alveolar glands which are lined by a single layer of columnar epithelial cells (Figure 2A). Cultures maintained in serum-supplemented medium without testosterone underwent epithelial atrophy, whereas the addition of $4\times10^{-6}$ M testosterone maintained the height and secretory activity of the alveolar epithelium and promoted epithelial cell proliferation (Figure 2B). Similar cultures treated with testosterone and $4\times10^{-5}$ M Estracyt (Figure 2C) or estramustine exhibited extensive necrosis of the alveolar epithelium, whereas treatment with $4\times10^{-5}$ M oestradiol-17$\beta$ caused marked epithelial atrophy (Figure 2D).

In serum-free medium, cultures maintained in the absence of testosterone were atrophic, whereas supplementation with $4\times10^{-6}$ M testosterone preserved the normal morphology and secretory activity of the epithelium (Figure 3A). Similar cultures treated with $4\times10^{-6}$ M testosterone and $4\times10^{-5}$ M Estracyt (Figure 3B) or estramustine also exhibited well-maintained alveolar epithelium. In contrast, treatment with $4\times10^{-5}$ M oestradiol-17$\beta$ was cytotoxic causing widespread necrosis of the alveolar epithelium and fibromuscular stroma (Figure 3C).

*Effects of concentration*

*I-UdR uptake* The effects of $4\times10^{-9}$, $4\times10^{-7}$ and $4\times10^{-5}$ M Estracyt, Emcyt, estramustine and oestradiol-17$\beta$ on the proliferative response of rat ventral prostate to $4\times10^{-7}$ M testosterone following four days of culture in serum-free medium are shown in Figure 4. At $4\times10^{-9}$ and $4\times10^{-7}$ M, oestradiol-17$\beta$ significantly ($P<0.01$) reduced the testosterone effect and was markedly inhibitory ($P<0.01$) at $4\times10^{-5}$ M. In contrast, the estramustine compounds were consistently less effective than oestradiol-17$\beta$ at all concentrations used.

Using the same procedure, Figure 5 demonstrates the effects of $4\times10^{-9}$, $4\times10^{-7}$ and $4\times10^{-5}$ M Estracyt, Emcyt, estramustine and oestradiol-17$\beta$ on the proliferative response to $4\times10^{-9}$ M testosterone, which is the minimum dose of testosterone for maximum stimulation of I-UdR uptake in serum-free cultures of rat ventral prostate (Mistry *et al.*, 1982). Treatment with $4\times10^{-9}$ and $4\times10^{-7}$ M oestradiol-17$\beta$ reduced the testosterone response to the level of the testosterone-free controls ($P<0.01$), whereas equimolar concentrations of the estramustine compounds were less effective. At $4\times10^{-5}$ M, Estracyt, Emcyt, and estramustine

Figure 3 Alveoli in explants of young adult rat ventral prostate cultured for 4 days in serum free-medium. H & E staining. (A) Explant treated with $4\times10^{-6}$ M testosterone showing actively secreting columnar epithelium ($\times$500). (B) Explant treated with $4\times10^{-6}$ M testosterone and $4\times10^{-5}$ Estracyt showing well-maintained alveolar epithelium ($\times$500). (C) Explant treated with $4\times10^{-6}$ M testosterone and $4\times10^{-5}$ M oestradiol-17$\beta$ showing extensive necrosis of the alveolar epithelium and fibromuscular stroma ($\times$500).

also reduced the testosterone response to control levels but treatment with $4\times10^{-5}$ M oestradiol-17$\beta$ remained more inhibitory. Pre-treatment with the estramustine compounds ($4\times10^{-5}$ M) for 48 h before the addition of $4\times10^{-9}$ testosterone did not enhance the inhibitory actions of these drugs, and oestradiol-17$\beta$ remained the most potent inhibitor of the testosterone response (Figure 6).

*Histology* Histologically, cultures treated with $4\times10^{-7}$ M testosterone alone or in combination with $4\times10^{-5}$ M Estracyt, Emcyt or estramustine in serum-free medium all showed well maintained, actively secreting alveolar epithelium. However, similar cultures treated with $4\times10^{-7}$ M testosterone and $4\times10^{-5}$ M oestradiol-17$\beta$ exhibited extensive epithelial and stromal necrosis.

Cultures treated with only $4\times10^{-9}$ M testosterone were well-preserved (Figure 7A), but the addition of $4\times10^{-5}$ M Estracyt (Figure 7B), Emcyt or estramustine caused epithelial atrophy. However, treatment with $4\times10^{-7}$ M oestradiol-17$\beta$ also produced severe epithelial regression (Figure 7C) and $4\times10^{-5}$ M oestradiol-17$\beta$ remained cytotoxic.

Figure 4 Effects of Estracyt (LS 299), Emcyt (LS 299Z), estramustine (LS 275) and oestradiol-17$\beta$ ($E_2$-17$\beta$) on $4\times10^{-7}$ M testosterone stimulated I-UdR uptake in serum free cultures of rat ventral prostate. Testosterone-free control cultures are represented by ■, medium only, and □, medium containing the alcohol diluent (mean ±s.e.; 16 explants/treatment group, 3 separate experiments). Dotted line indicates mean ±s.e. of stimulation index of testosterone only supplemented cultures.

Figure 5 Effects of $4\times10^{-5}$ M Estracyt (LS 299), Emcyt (LS 299Z), estramustine (LS 275) and oestradiol-17$\beta$ ($E_2$-17$\beta$) on $4\times10^{-9}$ M testosterone stimulated I-UdR uptake in serum-free cultures of rat ventral prostate. Testosterone-free control cultures are represented by ■, medium only, and □, medium containing the alcohol diluent (mean ±s.e.; 16 explants/treatment group, 3 separate experiments). Dotted line indicates mean ±s.e. of stimulation index of testosterone only supplemented cultures.

### Discussion

While several studies have demonstrated inhibitory effects of Estracyt on prostatic DNA synthesis (Forsberg & Hoisaeter, 1975; Hoisaeter, 1975, 1976*b*, 1977), others have been unable to distinguish the effect of Estracyt from that of oestradiol-17$\beta$ (Yamanaka *et al.*, 1977; Wakisaka *et al.*, 1979; Mistry *et*

**Figure 6** Proliferative responses of rat ventral prostate in serum-free medium following treatment with $4 \times 10^{-5}$ M Estracyt (LS 299), Emcyt (LS 299Z), estramustine (LS 275) and oestradiol-17$\beta$ ($E_2$-17$\beta$) for the first 48 h and $4 \times 10^{-9}$ M testosterone during the final 48 h of the culture. 'Control' represents testosterone-free cultures (mean $\pm$s.e.; 16 explants/treatment group, 3 separate experiments).

**Figure 7** Alveoli in explants of young adult rat ventral prostate cultured for 4 days in serum-free medium containing $4 \times 10^{-9}$ M testosterone. H & E staining. (A) Explant treated with $4 \times 10^{-9}$ M testosterone, showing well maintained alveolar epithelium ($\times$500). (B) Explant treated with $4 \times 10^{-9}$ M testosterone and $4 \times 10^{-5}$ M Extracyt showing low cuboidal alveolar epithelium ($\times$500). (C) Explant treated with $4 \times 10^{-9}$ M testosterone and $4 \times 10^{-7}$ oestradiol-17$\beta$ showing low cuboidal alveolar epithelium ($\times$500).

*al.*, 1983). In the present study, treatment with $4 \times 10^{-5}$ M Estracyt, estramustine and oestradiol-17$\beta$ in serum supplemented medium all had equally inhibitory effects on $4 \times 10^{-6}$ M testosterone induced I-UdR uptake, whereas in serum-free medium only oestradiol-17$\beta$ was effective in suppressing the response to testosterone. The histological results further demonstrated that the estramustine compounds had no antiprostatic effect in serum-free medium, but were cytotoxic in the presence of serum. In contrast, treatment with oestradiol-17$\beta$ was markedly cytotoxic in serum free medium, whereas it had an antiandrogenic effect causing epithelial atrophy in the presence of serum. Thus, serum appears to potentiate the antiprostatic activity of the estramustine compounds, while it reduces the inhibitory action of oestradiol-17$\beta$. The presence of steroid hormone binding proteins in the serum supplement may be responsible for eliminating the cytotoxic effect of oestradiol-17$\beta$ and reducing the magnitude of the inhibitory effect on I-UdR uptake by decreasing the concentration of free oestrogen in the medium. Similarly, the presence of steroid binding proteins may account for slight reductions in the stimulatory effect of testosterone on I-UdR uptake in serum supplemented cultures. However, the results did not indicate that increasing serum concentrations further affected the actions of either testosterone or oestradiol-17$\beta$.

In contrast to the present study, Hoisaeter (1975) found that in organ cultures of rat ventral prostate maintained in serum-supplemented medium (5% FCS) containing $4 \times 10^{-6}$ M testosterone and either $4 \times 10^{-5}$ M Estracyt or oestradiol-17$\beta$, only Estracyt had a significantly inhibitory effect on $^3$H-TdR uptake. Nevertheless, the histological results were remarkably similar to the present study, indicating that Estracyt had a pronounced cytotoxic effect on rat ventral prostate cultured in serum-supplemented medium while the retrogressive changes associated with oestradiol-17$\beta$ were less severe. Unlike the present study, however, the morphology of cultures maintained in the absence of exogenous testosterone was comparable to intact tissue and did not demonstrate epithelial atrophy typical of androgen deprivation (Hoisaeter, 1975). Moreover, Hoisaeter (1975) was unable to demonstrate any stimulation of $^3$H-TdR uptake in cultures treated with only $4 \times 10^{-6}$ M testosterone and this was attributed to a masking effect caused by endogenous hormones present in the serum supplement. While variations in the hormone content of different batches of foetal calf serum (Esber *et al.*, 1973) may be responsible for these contrasting results, the present study further suggests that serum supplementation obscures differences between the effect of oestradiol-17$\beta$ and its alkylated analogues.

Subsequent investigations using only serum-free medium showed that oestradiol-17$\beta$ remained consistently more effective than any of the estramustine compounds in suppressing testosterone induced I-UdR uptake. Oestradiol-17$\beta$ exhibited dose-related inhibitory effects on both $4 \times 10^{-7}$ and $4 \times 10^{-9}$ M testosterone stimulated I-UdR uptake, whereas the estramustine compounds were only inhibitory at $4 \times 10^{-5}$ M in cultures treated with physiological concentrations of testosterone (i.e. $4 \times 10^{-9}$ M). Pre-treatment (48 h) with the estramustine compounds did not enhance their ability to suppress the testosterone response, and their addition 48 h after testosterone reduced the magnitude of the inhibitory response. The histological results further demonstrated that treatment with $4 \times 10^{-9}$ M testosterone and any of the estramustine compounds at $4 \times 10^{-5}$ M caused epithelial atrophy, however lower concentrations of oestradiol-17$\beta$ produced comparable inhibition of I-UdR uptake and similar histological changes. Thus, the antiandrogenic effects of the estramustine compounds may be due to the release of some oestradiol-17β from the hormone cytotoxic complex. Although Hoisaeter (1975) did not detect any hydrolysis of Estracyt by rat ventral prostate in organ culture, more recently Symes & Milroy (1982) found that the cleavage rate of estramustine by prostatic tissue *in vitro* is low but it is nevertheless, dose dependent, being greater at $10^{-5}$ than $10^{-8}$ M. The inhibitory effects described with compounds in the range of $10^{-9}$–$10^{-8}$ M would be in the physiological range of concentrations. Steady state concentrations of estramustine in the plasma are of a similar range to those used in these experiments ($10^{-8}-10^{-7}$ M) (Hartley-Asp & Gunnarsson, 1982).

While the oestradiol-17β released upon hydrolysis of Estracyt probably contributes to the antiprostatic activity of this drug (Sandberg, 1983), a purely oestrogenic mode of action for Estracyt probably contributes to the antiprostatic activity of this drug (Sandberg, 1983), a purely oestrogenic mode of action for Estracyt has been discounted on evidence of anti-tumour activity in oestrogen relapsed prostatic cancer patients (Jonsson *et al.*, 1977; Madajewicz *et al.*, 1980; Leistenschneider & Nagel, 1980) and in oestrogen resistant animal tumours (Muntzing *et al.*, 1979). *In vitro* studies of hormone unresponsive prostatic cancer cells (DU 145) further suggest that estramustine is the biologically active form of Estracyt (Hartley-Asp & Gunnarsson, 1982; Hartley-Asp, 1984), however recent organ culture studies of human prostatic cancer tissue showed that DNA synthesis was equally inhibited by oestradiol-17β and estramustine (Mistry *et al.*, 1983). Moreover, the present *in vitro* system indicates that neither Estracyt nor its metabolite, estramustine, offer any selective advantage over that of their carrier-hormone, oestradiol-17β in suppressing testosterone-induced DNA synthesis in rat ventral prostate. Nevertheless, enhancement in the antiprostatic acitivty of the estramustine compounds in the presence of serum *in vitro* suggests that these drugs require biological activation for maximum efficacy.

### References

BJORK, P., FORSGREN, N., GUSTAFSSON, J.-A., POUSETTE, A. & HOGBERG, B. (1982). Partial characterization and 'quantitation' of human prostatic estramustine binding protein. *Cancer Res.*, **42**, 1935.

BUCHANAN, L.J. & RICHES, A.C. (1985). Proliferative responses of normal rat ventral prostate in organ culture: effects of testosterone and its metabolites in chemically defined medium. *Prostate*, **7**, 419.

BUCHANAN, L.J. & RICHES, A.C. (1986). Proliferative responses of rat ventral prostate: effects of variations in organ culture media and methodology. *Prostate* **8**, 63.

CHISHOLM, G.D. (1981). Perspectives and prospects. *Recent Results Cancer Res.*, **78**, 178.

EDSMYR, F., ANDERSON, L. & KONYVES, I. (1982). Estramustine phosphate (Estracyt): Experimental studies and clinical experience. In *Prostate Cancer*, G.H. Jacob & R. Hohenfellner (eds.), p. 253. Williams and Wilkins, Baltimore/London.

ESBER, H.J., PAYNE, I.J. & BOGDEN, A.E. (1973). Variability of hormone concentration and ratios in commercial sera used for tissue culture. *J. Natl Cancer Inst.*, **50**, 559.

FLANDERS, D.W. (1984). Review: Prostate Cancer Epidemiology. *Prostate*, **5**, 621.

FORSBERG, J.G. & HOISAETER, P.A. (1975). Effects of hormone-cytostatic complexes on the rat ventral prostate *in vivo* and *in vitro*. *Vitamins and Hormones*, **33**, 137.

FORSGREN, B., BJORK, P., CARLSTROM, K., GUSTAFSSON, J.-A., POUSETTE, A. & HOGBERG, B. (1979). Purification and distribution of a major protein in rat prostate that binds estramustine, a nitrogen mustard derivative of estradiol-17. *Proc. Nat. Acad. Sci. USA*, **76**, 3149.

FORSGREN, B., BJORK, P., CARLSTROM, K., GUSTAFSSON, J.-A., POUSETTE, A. & HOGBERG, B. (1981). The presence in rat and human prostate of proteins that bind steroid-cytostatic complexes. In *The Prostatic Cell: Structure and Function*, p. 391. Alan R. Liss, Inc., New York.

HARTLEY-ASP, B. (1984). Estramustine induced mitotic arrest in two human prostatic carcinoma cell lines DU 145 and PC-3. *Prostate* **5**, 93.

HARTLEY-ASP, B. & GUNNARSSON, P.O. (1982). Growth and cell survival following treatment with estramustine, nor-nitrogen mustard, estradiol and testosterone of a human prostatic cancer cell line (DU 145). *J. Urol.*, **127**, 818.

HOISAETER, P.A. (1975). Incorporation of $^{3}$H-thymidine into rat ventral prostate in organ culture. Influence of hormone-cytostatic complexes. *Invest. Urol.*, **12**, 479.

HOISAETER, P.A. (1976*a*). Studies on the conversion of estradiol linked to a cytostatic agent (Estracyt$^{R}$) in various rat tissues. *Acta Endocrinol.*, **82**, 661.

HOISAETER, P.A. (1976*b*). Incorporation of $^{3}$H-thymidine and $^{14}$C-amino acids into the ventral prostate after *in vivo* treatment with estradiol-3N-bis (2 cholorethyl) carbamate-17β-phosphate (Estracyt) and its estrogen and cytostatic parts. *Invest. Urol.*, **14**, 85.

HOISAETER, P.A. (1977). Effect of steroid alkylating agent (Estramustine phosphate) in rat ventral prostate. *Scand. J. Urol. Nephrol.*, Suppl. **46**, 72.

HOISAETER, P.A. (1984). Mode of action of Estracyt. *Urology*, **XIII** (Suppl.), 46.

HOISEATER, P.A. & BAKKE, A. (1983). Estramustine phosphate (Estracyt$^{R}$): Experimental and clinical studies in Europe. *Sem. Oncol.*, **X**, Suppl. 3, 27.

JONSSON, G., HOGBERG, B. & NILSSON, T. (1977). Treatment of advanced prostatic carcinoma with estramustine phosphate (Estracyt$^{R}$). *Scand. J. Urol. Nephrol.*, **11**, 231.

JONSSON, G. & HOGBERG, B. (1971). Treatment of advanced prostatic carcinoma with Estracyt$^{R}$. *Scand. J. Urol. Nephrol.*, **5**, 103.

LEISTENSCHNEIDER, W. & NAGEL, R. (1980). Estracyt therapy of advanced prostatic cancer with special reference to control therapy with cytology and DNA cytophotometry. *Eur. Urol.*, **6**, 111.

MADAJEWICZ, S., CATANE, R., MITTLEMAN, R., WAJSMAN, Z. & MURPHY, G.P. (1980). Chemotherapy of advanced, hormonally resistant prostatic carcinoma. *Oncol.*, **37**, 53.

MISTRY, D., BUCHANAN, L., DATTANI, G., WEAVER, P. & RICHES, A.C. (1982). Proliferative responses of cultured rat prostate and human benign prostatic hyperplasia. *Prostate*, **3**, 291.

MISTRY, D., WEAVER, J.P. & RICHES, A.C. (1983). Organ culture studies of human prostatic adenocarcinomas. *Prostate*, **4**, 307.

MUNTZING, J., JENSEN, G. & HOGBERG, E. (1979). Pilot study on the growth inhibition by estramustine phosphate (Estracyt) of rat mammary tumours sensitive and insensitive to oestrogens. *Acta Pharmacol. Toxicol.*, **44**, 1.

MURPHY, G.P., SLACK, N.H. & MITTELMAN, A. (1983). Experiences with estramustine phosphate (Estracyt$^{R}$, Emcyt$^{R}$) in prostate cancer. *Sem. Oncol.*, **X**, Suppl. 3, 34.

NILSSON, T. & JONSSON, G. (1977). Estramustine phostatic carcinoma. *Current Chemotherapy*, **2**, 1282.

PLYM-FORSHELL, G. & NILSSON, H. (1974). Administration of labelled estramustine phosphate (Estracyt$^{R}$), estradiol-17β-phosphate and estradiol to rats. *Acta Pharmacol. Toxicol.*, **35**, 28.

PLYM-FORSHELL, G., MUNTZING, J., LINDSTEDT, E. & DENEKER, H. (1976). The absorption, metabolism and excretion of Estracyt in patients with prostatic carcinoma. *Invest. Urol.*, **14**, 128.

RICHES, A.C., GORE, D., DOCHERTY, J. & LITTLEWOOD, V. (1976). The use of 5-[$^{125}$I]-iodo-2'-deoxyuridine for monitoring DNA synthesis in organ culture. *J. Anat.*, **121**, 323.

SANDBERG, A. (1983). Metabolic aspects and actions unique to Estracyt$^{R}$. *Sem. Oncol.*, **X**, Suppl. 3, 3.

SYMES, E.K. & MILROY, E.J. (1982). The synthesis of $^{3}$H-labelled steroid-nitrogen mustard derivatives and studies on their action in rat and human prostrate. *J. Steroid Biochem.*, **17**, 23.

TEW, K.D. (1983). The mechanism of action of estramustine. *Sem. Oncol.*, **X**, Suppl. 3, 21.

WALZER, Y., OSWALT, J. & SOLOWAY, M. (1984). Estramustine phosphate – hormone, chemotherapeutic agent, or both? *Urology*, **XXIV**, 53.

WAKISAKA, M., IWASAKI, I. & CHIMAZSAKI, J. (1979). Effect of estramustine phosphate (Estracyt) on transplantable mouse tumours. *Urology Res.*, **7**, 291.

YAMANAKA, H., SHIMAZAKI, J., IAMI, K., SUGIYAMA, Y. & SHIDA, K. (1977). Effect of Estracyt on the rat prostate. *Invest. Urol.*, **14**, 400.